# Real-World outcomes of intravitreal treatment switching in retinal diseases: Analysis of 3,038 treated cases

**DOI:** 10.1177/11206721261471209

**Published:** 2026-07-23

**Authors:** Tugce Horozoglu Ceran, Gullu Jabbarova, Rabia Yokus, Muberra Akdogan, Mustafa Dogan

**Affiliations:** 1 Department of Ophthalmology, Faculty of Medicine, 534521 Afyonkarahisar Health Sciences University, Afyonkarahisar, Turkiye; 2 Department of Ophthalmology, 652493 Ministry of Health Rize State Hospital, Rize, Turkiye

**Keywords:** Aflibercept, anti-VEGF, diabetic macular edema, intravitreal injection, neovascular age-related macular degeneration, treatment switching

## Abstract

**Purpose:**

To evaluate the frequency and patterns of intravitreal therapy (IVT) switching in retinal diseases and describe associated visual acuity outcomes in real-world clinical practice.

**Materials and Methods:**

This retrospective, monocentric study evaluated consecutive IVT-treated cases at a tertiary retina unit. Demographic data, diagnoses, initial agent, injection number, and treatment switches were recorded. Best-corrected visual acuity (BCVA, LogMAR) was assessed at baseline and final follow-up. Switching was defined as a change to another anti-VEGF agent or intravitreal dexamethasone (DEX) implant for inadequate functional and/or anatomical response. BCVA changes were compared using appropriate tests, stratified by indication (diabetic macular edema [DME], neovascular age-related macular degeneration [nAMD], retinal vein occlusion [RVO]).

**Results:**

A total of 3,038 IVT-treated cases (11,345 injections) were analyzed. The main indications were DME (55.5%), nAMD (36.4%), and RVO (6.0%). Overall, 22.9% of treated cases underwent treatment switching, most frequently in DME (24.8%) and nAMD (20.8%). Within-group analyses showed significant BCVA improvement after switching in DME (p = 0.001), RVO (p = 0.032), and nAMD (p = 0.009), while non-switched groups showed no significant change. Aflibercept was the most commonly selected second-line agent (81.7%), and the aflibercept subgroup showed significant within-group BCVA improvement (*p* < 0.001). Because treatment choice was not randomized, this finding should not be interpreted as evidence of superiority over other second-line agents.

**Conclusions:**

In this retrospective real-world cohort, treatment switching was common in routine retinal practice, and switched cases showed within-group BCVA improvement in some diagnostic subgroups. These findings should be interpreted as descriptive associations rather than evidence of a causal treatment effect.

## Introduction

Over the past decade, intravitreal therapy has become a cornerstone in the management of retinal diseases.^1^ Anti-vascular endothelial growth factor (anti-VEGF) agents are the first-line treatment for neovascular age-related macular degeneration (nAMD)^1^ and are also widely used for macular edema secondary to diabetic macular edema (DME) and retinal vein occlusion (RVO).^2^ Anti-VEGF agents, including bevacizumab, ranibizumab, and aflibercept, reduce macular edema and suppress neovascular activity, thereby helping preserve or improve best-corrected visual acuity (BCVA). In selected cases of DME and RVO, an intravitreal dexamethasone (DEX) implant may also be considered as an alternative treatment option.^2^

Despite their proven efficacy, a substantial proportion of eyes show an incomplete or suboptimal response to initial anti-VEGF therapy, particularly in DME.^3^ Inadequate response or the development of tachyphylaxis, defined as a progressive reduction in therapeutic effect with repeated or prolonged drug exposure, may lead clinicians to consider a change in treatment strategy.^4^ Previous studies have suggested that switching to another anti-VEGF agent or to an intravitreal corticosteroid may be associated with anatomical and/or functional benefit in selected treatment-resistant cases.^5,6^

In this retrospective real-world study, we evaluated intravitreal treatment patterns, injection burden, switching frequency, and visual acuity outcomes at a tertiary retina unit. We also described the distribution of second-line treatment choices used in routine clinical practice.

## Materials and methods

### Study design

This study was designed as a calendar-year real-world analysis of IVT-treated cases managed during 2024. This was a monocentric, retrospective, real-world observational study. The study protocol was approved by the Clinical Research Ethics Committee of Afyonkarahisar Health Sciences University (July 04, 2025, 2025/9) and was conducted in accordance with the Declaration of Helsinki. Owing to the retrospective design, additional written informed consent was not required.

### Patient selection

All consecutive IVT-treated retinal disease cases managed in our retina unit between January 2024 and December 2024 (12 months) were eligible and included. No strict exclusion criteria were applied in order to reflect routine real-world clinical practice. Diagnoses were established by retina specialists on the basis of clinical examination and multimodal retinal imaging, including fundus fluorescein angiography (FFA), optical coherence tomography (OCT), and optical coherence tomography angiography (OCTA), when indicated.

### Treatment and follow-up

For each case, baseline BCVA was defined as the first recorded BCVA value in 2024, corresponding to the first IVT-related visit during the study year. Final BCVA was defined as the last recorded BCVA value in 2024, corresponding to the last available follow-up visit during the same calendar year. Treatment switching was recorded if a change in intravitreal agent occurred at any time during 2024. Therefore, BCVA outcomes represent changes between the first and last available BCVA measurements within the 2024 study period, rather than fixed-interval pre-switch and post-switch visual acuity outcomes.

In the present study, treatment switching was defined as any change from the initially administered intravitreal agent to another therapeutic agent during follow-up. This included both anti-VEGF-to-anti-VEGF switches, such as bevacizumab to aflibercept or ranibizumab, and anti-VEGF-to-DEX implant switches. Because of the retrospective real-world design, switching was not based on a prespecified protocolized threshold. In routine practice, the decision to switch was made after repeated exposure to the initial agent when insufficient treatment response was documented by the treating retina specialist. Insufficient response was assessed using both functional and anatomical findings, including lack of BCVA improvement or BCVA deterioration, persistent or worsening intraretinal/subretinal fluid, persistent macular edema or increased central macular thickness on OCT, and persistent disease activity on clinical examination and/or angiographic imaging when available. Anti-VEGF-to-anti-VEGF and anti-VEGF-to-DEX switches were recorded separately; however, the primary analysis evaluated all switched cases together, with additional descriptive subgroup analyses according to the second-line agent.

### Data collection and assessment

Clinical data were retrospectively collected from electronic medical records and patient files. Recorded parameters included demographic characteristics (age, sex), baseline retinal diagnosis, the specific intravitreal agents administered, total number of injections, and treatment switching. The present study was designed to evaluate treatment switching frequency, switching patterns, and associated BCVA outcomes in a large real-world IVT cohort. Although OCT findings and detailed injection histories were available in the clinical records and were used in routine decision-making, quantitative OCT outcomes and fixed pre-switch/post-switch injection counts were not included as formal study endpoints. This decision was made to maintain a focused analysis of switching patterns and visual outcomes across a large heterogeneous cohort.

BCVA was extracted for each case. Snellen visual acuity measurements were converted to logarithm of the minimum angle of resolution (logMAR) values for statistical analysis. Baseline BCVA, defined as the first recorded BCVA value during 2024, and final BCVA, defined as the last recorded BCVA value during 2024, were recorded for all included cases.

Anti-VEGF-to-anti-VEGF switches and anti-VEGF-to-DEX implant switches were recorded as distinct switch categories. The primary switching analysis included all switches together, whereas additional descriptive subgroup analyses were performed according to the second-line agent, including anti-VEGF agents and DEX implant.

### Statistical analysis

Given the retrospective observational design, statistical analyses were primarily descriptive and exploratory. Statistical analysis was performed using IBM SPSS Statistics version 25.0 (IBM Corp., Armonk, NY, USA). Normality of continuous variables was assessed using the Kolmogorov-Smirnov test. Normally distributed variables were summarized as mean ± standard deviation, whereas non-normally distributed variables were presented as median (minimum−maximum). Baseline and final BCVA data were available for all 3,038 cases included in the study. Therefore, no cases were excluded from the BCVA outcome analyses because of missing baseline or final BCVA data. Baseline and final BCVA values were compared using a paired Student's t-test for parametric data or the Wilcoxon signed-rank test for non-parametric data. Comparisons between switched and non-switched cases were performed using the independent samples t-test (parametric) or Mann-Whitney U test (non-parametric) for continuous variables. Categorical variables were compared using the chi-square test. A two-sided *p*-value < 0.05 was considered statistically significant for all analyses.

## Results

A total of 3,038 IVT-treated cases recorded in 2024 were included in the analysis. The mean age was 67.9 ± 9.8 years, with a median age of 68 years (range, 22−103 years). Of these cases, 54.3% were female (n = 1,650) and 45.7% were male (n = 1,388). DME/DRP was the most common indication for treatment (55.5%; n = 1,687), followed by nAMD (36.4%; n = 1,105) and macular edema secondary to RVO (6.0%; n = 183). Less common indications, including ERM, uveitic macular neovascularization, Irvine-Gass syndrome, and myopic macular neovascularization, accounted for approximately 2% of cases (Table 1).

**Table 1.** Patients’ demographics and distribution of retinal diagnoses.

Parameters	*n*	%
Male	1388	45.7
Female	1650	54.3
DME/DRP	1687	55.5
ERM	46	1.5
RVO	183	6.0
nAMD	1105	36.4
Myopic macular neovascularization	1	0.0
Irvine-Gass syndrome	1	0.0
Uveitic macular neovascularization	15	0.5
Total	3038	100.0

*DME/DRP: diabetic macular edema/diabetic retinopathy; ERM: epiretinal membrane; RVO: retinal vein occlusion; nAMD: neovascular age-related macular degeneration.

During the study period, a total of 11,345 intravitreal injections were administered. The mean number of injections per case was 3.73, with a median of 4 injections (range, 1–10). The initial treatment agents were bevacizumab in 51.8% of cases (n = 1,575), aflibercept in 31.0% (n = 941), DEX implant in 10.8% (n = 328), and ranibizumab in 6.4% (n = 194). Treatment exposure in the present analysis was summarized as the total number of intravitreal injections administered during the 2024 calendar-year observation period.

The frequency of treatment switching varied by diagnosis. Less common diagnoses were retained in the switching analysis and grouped consistently with Table 1 where appropriate. Switching occurred in 24.8% of DME/DRP cases (418/1,687), 21.9% of RVO cases (40/183), and 20.8% of nAMD cases (230/1,105). Switching frequencies for less common diagnostic groups are presented in Table 2. Overall, 22.9% of cases (n = 695) underwent treatment switching, whereas 77.1% (n = 2,343) did not.

**Table 2.** Treatment switching rates by diagnostic group.

Diagnosis	No switch n (%)	Switch n (%)	Total n	p
DME/DRP	1269 (75.2%)	418 (24.8%)	1687	0.016
ERM	39 (84.8%)	7 (15.2%)	46	0.016
RVO	143 (78.1%)	40 (21.9%)	183	0.016
nAMD	875 (79.2%)	230 (20.8%)	1105	0.016
Uveitic macular neovascularization	15 (100.0%)	0 (0.0%)	15	0.016
Myopic macular neovascularization	1 (100.0%)	0 (0.0%)	1	0.016
Irvine-Gass syndrome	1 (100.0%)	0 (0.0%)	1	0.016
Total	2343 (77.1%)	695 (22.9%)	3038	

* DME/DRP: diabetic macular edema/diabetic retinopathy; ERM: epiretinal membrane; RVO: retinal vein occlusion; nAMD: neovascular age-related macular degeneration. Other retinal diseases included Irvine-Gass syndrome, myopic macular neovascularization, and uveitic macular neovascularization.

Visual acuity outcomes were evaluated as within-group changes in logMAR BCVA. Baseline and final BCVA data were available for all 3,038 cases; therefore, no case was excluded from the BCVA analysis because of missing BCVA data. Because the study was structured as a calendar-year real-world analysis, baseline and final BCVA values corresponded to the first and last recorded BCVA measurements during 2024. Thus, BCVA changes reflect the visual acuity difference between these two time points within the study year. Follow-up timing and the timing of treatment switching were not standardized, but reflected routine clinical practice. In the DME/DRP group, non-switched cases showed no significant change (baseline 0.8435 ± 0.5848 vs final 0.8440 ± 0.5949; *p* = 0.836), whereas switched cases showed a statistically significant within-group improvement (baseline: 0.8803 ± 0.6075 vs. final 0.8125 ± 0.5436; *p* = 0.001). In the ERM group, non-switched cases showed a borderline worsening (baseline: 0.7217 ± 0.3908 vs. final 0.8503 ± 0.4358; *p* = 0.050), whereas switched cases showed no significant difference (baseline: 1.0368 ± 0.5815 vs final 1.0559 ± 0.6288; *p* = 1.000). In RVO, non-switched cases exhibited a near-significant improvement (baseline: 1.0209 ± 0.7444 vs final 0.9578 ± 0.6878; *p* = 0.053), while switched cases showed a statistically significant within-group improvement (baseline: 1.0974 ± 0.7092 vs final 0.9540 ± 0.7740; *p* = 0.032). In the nAMD group, non-switched cases showed no significant change (baseline 1.1802 ± 0.7701 vs final 1.1944 ± 0.7406; *p* = 0.276), whereas switched cases showed a statistically significant within-group improvement (baseline 1.2573 ± 0.7765 vs final 1.1790 ± 0.7132; *p* = 0.009). In the uveitic macular neovascularization group, non-switched cases did not show a statistically significant difference (baseline: 0.8475 ± 0.4766 vs final 0.6934 ± 0.3687; *p* = 0.166) (Table 3).

**Table 3.** Visual acuity (LogMAR) changes by diagnosis and switch status.

Diagnosis	Switch	Baseline mean ± SD	Final mean ± SD	p
DME/DRP	No	0.8435 ± 0.5848	0.8440 ± 0.5949	0.836
DME/DRP	Yes	0.8803 ± 0.6075	0.8125 ± 0.5436	0.001
ERM	No	0.7217 ± 0.3908	0.8503 ± 0.4358	0.050
ERM	Yes	1.0368 ± 0.5815	1.0559 ± 0.6288	1.000
RVO	No	1.0209 ± 0.7444	0.9578 ± 0.6878	0.053
RVO	Yes	1.0974 ± 0.7092	0.9540 ± 0.7740	0.032
Uveitic macular neovascularization	No	0.8475 ± 0.4766	0.6934 ± 0.3687	0.166
nAMD	No	1.1802 ± 0.7701	1.1944 ± 0.7406	0.276
nAMD	Yes	1.2573 ± 0.7765	1.1790 ± 0.7132	0.009

*DME/DRP: diabetic macular edema/diabetic retinopathy; ERM: epiretinal membrane; RVO: retinal vein occlusion; nAMD: neovascular age-related macular degeneration.

Across all indications combined, non-switched cases showed no significant change in BCVA (baseline logMAR 0.9783 ± 0.6859 (median 0.7959; Q1 = 0.4983; Q3 = 1.5051) vs. final 0.9805 ± 0.6758 (median 0.7959; Q1 = 0.4983; Q3 = 1.5051); *p* = 0.849). Switched cases, by contrast, showed a statistically significant within-group improvement (baseline 1.0195 ± 0.6949 (median 0.7959; Q1 = 0.4983; Q3 = 1.5051) vs final 0.9444 ± 0.6374 (median 0.6990; Q1 = 0.4983; Q3 = 1.5051); *p* < 0.001) (Table 4).

**Table 4.** Comparison of visual acuity changes between switch and non-switch groups.

Group	BCVA (LogMAR) mean ± SD	Median	Q1 (25p)	Q3 (75p)	p
No switch (Baseline)	0.9783 ± 0.6859	0.7959	0.4983	1.5051	0.849
No switch (Final)	0.9805 ± 0.6758	0.7959	0.4983	1.5051	
Switch (Baseline)	1.0195 ± 0.6949	0.7959	0.4983	1.5051	<0.001
Switch (Final)	0.9444 ± 0.6374	0.6990	0.4983	1.5051	

When visual outcomes were stratified by second-line agent, among the switched cases, the aflibercept subgroup showed a significant within-group BCVA improvement (baseline 1.0199 ± 0.6858 (median 0.7959; Q1 = 0.5242; Q3 = 1.5051) vs. final 0.9401 ± 0.6273 (median 0.7474; Q1 = 0.4983; Q3 = 1.5051); *p* < 0.001). After switching to bevacizumab, no statistically significant within-group change was observed (baseline 1.6344 ± 1.4024 (median 1.5051; Q1 = 0.3010; Q3 = 3.0969) vs. final 1.9333 ± 1.1032 (median 1.7959; Q1 = 0.9031; Q3 = 3.0969); *p* = 0.180). In the DEX implant subgroup (n = 85), no significant change in BCVA was observed (baseline: 0.9395 ± 0.6408 (median 0.7959; Q1 = 0.3979; Q3 = 1.5051) vs final 0.9308 ± 0.6409 (median 0.6990; Q1 = 0.4983; Q3 = 1.5051); *p* = 0.931). In the ranibizumab subgroup (n = 39), BCVA showed a borderline improvement (baseline 1.1502 ± 0.8742 (median 0.9515; Q1 = 0.3979; Q3 = 1.5778) vs final 0.9702 ± 0.7117 (median 0.6505; Q1 = 0.3979; Q3 = 1.7959); *p* = 0.049) (Table 5).

**Table 5.** Statistical significance of visual acuity change by second-line agent.

Group	BCVA (LogMAR) mean ± SD	Median	Q1 (25p)	Q3 (75p)	*p*
Aflibercept (Pre-treatment)	1.0199 ± 0.6858	0.7959	0.5242	1.5051	<0.001
Aflibercept (Post-treatment)	0.9401 ± 0.6273	0.7474	0.4983	1.5051	
Bevacizumab (Pre-treatment)	1.6344 ± 1.4024	1.5051	0.3010	3.0969	0.180
Bevacizumab (Post-treatment)	1.9333 ± 1.1032	1.7959	0.9031	3.0969	
Dexamethasone (Pre-treatment)	0.9395 ± 0.6408	0.7959	0.3979	1.5051	0.931
Dexamethasone (Post-treatment)	0.9308 ± 0.6409	0.6990	0.4983	1.5051	
Ranibizumab (Pre-treatment)	1.1502 ± 0.8742	0.9515	0.3979	1.5778	0.049
Ranibizumab (Post-treatment)	0.9702 ± 0.7117	0.6505	0.3979	1.7959	

After switching, aflibercept was the most commonly chosen second-line agent, administered in 81.7% of switched cases (568/695). DEX implant was preferred in 12.2% (85/695), and ranibizumab in 5.6% (39/695) (Table 6). When switch type was considered, most switches represented anti-VEGF-to-anti-VEGF changes, whereas anti-VEGF-to-DEX implant switching accounted for a smaller subgroup. Outcomes were therefore presented both for the overall switched cohort and descriptively according to the second-line agent.

**Table 6.** Cross-distribution of intravitreal agent switching.

Initial agent	To aflibercept n (%)	To bevacizumab n (%)	To dexamethasone n (%)	To ranibizumab n (%)	Total n (%)
Aflibercept	0 (0.0)	1 (4.3)	21 (91.3)	1 (4.3)	23 (100.0)
Bevacizumab	540 (84.9)	0 (0.0)	60 (9.4)	36 (5.7)	636 (100.0)
Dexamethasone	27 (87.1)	2 (6.5)	0 (0.0)	2 (6.5)	31 (100.0)
Ranibizumab	1 (20.0)	0 (0.0)	4 (80.0)	0 (0.0)	5 (100.0)
Total	568 (81.7)	3 (0.4)	85 (12.2)	39 (5.6)	695 (100.0)

## Discussion

Previous studies suggest that switching therapy in eyes with an inadequate response to first-line anti-VEGF treatment may be associated with visual and/or anatomical benefit in selected cases.^7^ In DME, eyes with a suboptimal response to bevacizumab may show additional visual improvement after switching to ranibizumab or aflibercept during follow-up.^7^ The SWIRL study by Louis et al., based on real-world data, suggested that eyes with bevacizumab-refractory DME may show functional improvement after switching, and that both ranibizumab and aflibercept may be used as second-line options.^7^ Similarly, a systematic review of refractory DME reported that switching to aflibercept was associated with a reduction in central macular thickness and improvement in edema parameters after the first three injections.^5^ Other series have also suggested that switching to ranibizumab may provide anatomical benefit, particularly with respect to central retinal thickness.^5^ Taken together, the available literature indicates that switching anti-VEGF agents in treatment-resistant DME may be associated with anatomical benefit and, in some cases, functional improvement.

Most published studies have evaluated switching within a single indication. In nAMD, switching from ranibizumab or bevacizumab to aflibercept is commonly considered in eyes with persistent subretinal or intraretinal fluid. Several small studies have described anatomical improvement on OCT after such a switch, whereas visual acuity gains have been less consistent.^8^ For example, in a 42-month follow-up study of 33 eyes switched from ranibizumab to aflibercept, central macular thickness decreased substantially, but visual acuity was largely maintained rather than markedly improved.^8^ larger registry data also indicate that treatment switching is common in routine practice. In the Dutch FRB registry, 70.9% of eyes initially treated with bevacizumab were later switched, most commonly to aflibercept or ranibizumab, after a median of approximately 12 months.^9^ These findings suggest that switching represents an established real-world management strategy in nAMD, although the magnitude of functional benefit may vary across cohorts.^9^

In macular edema secondary to RVO, intravitreal corticosteroid implants are frequently considered as an alternative option in eyes with insufficient response to anti-VEGF therapy. Several studies have reported that switching to Ozurdex may reduce edema and may be associated with short-term improvement in both macular thickness and visual acuity, although repeated injections may be required to maintain these effects.^10^ In addition, a recent meta-analysis suggested that earlier versus later switching to DEX may be associated with different anatomical outcomes in DME and RVO.^11^ Because our study did not include a standardized anatomical outcome analysis, these reports are best interpreted as external context rather than direct confirmation from our cohort. Nonetheless, they highlight that switching strategies may differ across retinal diseases and according to the relative weight of functional and anatomical treatment goals.

Few studies have directly compared different second-line agents. Current evidence suggests that aflibercept is frequently selected as a second-line treatment, particularly in refractory DME.^5,12^ Some reports have suggested that switching to aflibercept may be associated with anatomical dryness not achieved with ranibizumab or bevacizumab in selected eyes.^5^ A meta-analysis also suggested that, in DME resistant to prior anti-VEGF therapy, aflibercept may be more likely to achieve complete macular drying than some alternative agents.^12^ However, switching to ranibizumab, including biosimilars, has also been reported to stabilize disease activity in selected settings.^13^ Importantly, the SWIRL study suggested that both ranibizumab and aflibercept may be useful second-line options in DME, without establishing clear superiority of one over the other.^7^

When an intravitreal DEX implant is considered as a second-line approach, the pattern may differ from that of anti-VEGF-to-anti-VEGF switching. Previous studies have reported that DEX implantation may reduce edema in eyes with DME or RVO that respond insufficiently to anti-VEGF therapy, although visual acuity improvement may be less consistent.^14^ A meta-analysis suggested that dexamethasone implants can provide meaningful anatomical benefit, while the corresponding visual improvement may be limited, potentially because of factors such as cataract progression.^14^ In this context, DEX may remain a reasonable option in selected patients, including pseudophakic eyes, eyes with a suspected inflammatory component, or patients who may have difficulty adhering to frequent injection schedules.^14^

Long-term real-world data indicate that switching therapy is not an uncommon event but rather a frequent part of retinal disease management. In the 5-year nAMD registry analysis, over half of eyes initially treated with bevacizumab were eventually switched to another anti-VEGF agent, often within the first 12–18 months.^9^ These findings suggest that a meaningful proportion of eyes in routine practice show a suboptimal response to first-line therapy, prompting a change in treatment strategy. However, many published reports remain limited by relatively small sample sizes. For example, the 42-month nAMD series evaluating ranibizumab-to-aflibercept switching included only 33 eyes.^8^ Similarly, many DME switching studies have been single-center analyses with limited sample sizes. In this context, our cohort of 3,038 IVT-treated cases represents a relatively large real-world dataset evaluating switching patterns and associated visual acuity outcomes.

Unlike many previously published studies that focused on a single indication, our analysis included multiple retinal diseases within the same tertiary retina practice. This made it possible to examine both shared and disease-specific patterns. In our cohort, switched cases with DME showed a significant within-group visual improvement during follow-up, whereas non-switched DME cases showed no significant change. Switched cases with RVO also showed a significant within-group improvement. In nAMD, switched cases likewise showed a modest but statistically significant within-group visual improvement during follow-up, whereas non-switched nAMD cases did not show a significant change. These findings suggest that the visual acuity trajectory after switching may differ by indication, although all such comparisons should be interpreted cautiously in view of the retrospective observational design and the absence of adjustment for baseline treatment-selection factors.

Our findings also reflect the treatment choices made in daily practice. In our cohort, aflibercept was by far the most frequently selected second-line agent. The aflibercept subgroup showed a significant within-group improvement in BCVA during follow-up, whereas no significant within-group change was observed in the bevacizumab or DEX subgroups, and the ranibizumab subgroup showed a borderline result. These observations are consistent with prior reports suggesting that aflibercept is commonly selected after inadequate response to initial therapy, particularly in DME.^5,12^ Although the aflibercept subgroup showed significant within-group BCVA improvement, second-line treatment choice was not randomized and was determined by clinical judgment. Therefore, these findings should be interpreted as descriptive within-group observations and should not be considered evidence of comparative superiority of aflibercept over other second-line agents.

Several methodological considerations should be acknowledged when interpreting these findings. First, because treatment switching was performed according to the treating physician's clinical judgment rather than random allocation or a prespecified protocol, switched and non-switched cases may have differed in disease activity, anatomical response, baseline visual status, treatment burden, and follow-up characteristics. Therefore, the observed visual acuity changes should be interpreted as descriptive real-world associations rather than causal effects of switching. Within-group BCVA improvement observed in some switched subgroups may partly reflect baseline disease severity, regression to the mean, treatment intensity, or other unmeasured clinical factors. Future prospective studies with standardized switching criteria and adjusted or propensity-matched analyses are needed to better estimate the independent effect of switching.

Second, follow-up timing was not standardized. This study was designed as a calendar-year real-world analysis, and BCVA outcomes were based on the first and last recorded BCVA measurements within 2024. Therefore, the intervals from baseline to switching and from switching to final BCVA assessment varied between cases and were not used as fixed analytical time points. This should be considered when interpreting the visual acuity findings as post-switch outcomes.

Finally, although OCT findings and detailed treatment histories were used in routine clinical decision-making, standardized quantitative OCT outcomes and fixed pre-/post-switch injection counts were not included as formal endpoints of the present analysis.

Real-world evidence shows that achieving the injection frequencies typically reported in randomized clinical trials is often difficult in everyday practice. In our cohort, the mean number of injections over 12 months was 3.73, which is lower than the frequency commonly described in controlled trial settings. This finding is consistent with other real-world reports. Factors such as treatment adherence, procedural anxiety, the logistical burden of repeated hospital visits, and financial or organizational barriers may all contribute to lower treatment intensity. Large datasets, including the AURA study, suggest that lower injection frequencies may adversely affect the durability of visual gains.^15^ Therefore, our results should also be interpreted in the context of real-world undertreatment, which may influence visual outcomes independently of switching strategy.

Overall, our real-world dataset addresses a practical clinical question: how are eyes with an inadequate response to initial intravitreal therapy managed in routine practice, and what visual acuity changes are observed after switching? In this cohort, switching was common, particularly in DME and nAMD, and some subgroups showed favorable within-group BCVA changes during follow-up. However, because this was a retrospective observational study and switching was based on clinician judgment, the findings should be interpreted as descriptive associations rather than evidence of comparative efficacy or optimal timing. These results nevertheless support the importance of flexible, individualized treatment planning in routine retinal practice.

## Conclusions

In cases with an inadequate response to initial intravitreal therapy, switching to an alternative agent was a common strategy in routine clinical practice. In our cohort, aflibercept was the most frequently selected second-line agent, and the aflibercept subgroup showed a significant within-group BCVA improvement during follow-up. However, because treatment switching and second-line agent selection were driven by clinical indication and disease activity rather than randomization, these findings should be interpreted as descriptive real-world associations. They should not be considered evidence that switching itself caused the observed visual improvement or that aflibercept is superior to other second-line agents.
